# First Report of the Detection of DENV1 in Human Blood Plasma with Near-Infrared Spectroscopy

**DOI:** 10.3390/v14102248

**Published:** 2022-10-13

**Authors:** Brendon Goh, Paul Visendi, Anton R. Lord, Silvia Ciocchetta, Wenjun Liu, Maggy T. Sikulu-Lord

**Affiliations:** 1 School of Biological Sciences, Faculty of Science, The University of Queensland, Brisbane 4067, Australia; 2 School of Biology & Environmental Science, Faculty of Science, Queensland University of Technology, Brisbane 4000, Australia; 3 Centre for Data Science, School of Computer Science, Queensland University of Technology, Brisbane 4000, Australia; 4 UQ Spatial Epidemiology Laboratory, School of Veterinary Science, The University of Queensland, Brisbane 4113, Australia; 5 Australian Defence Force, Malaria and Infectious Disease Institute, Brisbane 4051, Australia

**Keywords:** near-infrared spectroscopy, dengue, arbovirus, diagnosis, machine learning, artificial neural networks

## Abstract

Dengue virus (DENV) is the world’s most common arboviral infection, with an estimated 3.9 million people at risk of the infection, 100 million symptomatic cases and 10,000 deaths per year. Current diagnosis for DENV includes the use of molecular methods, such as polymerase chain reaction, which can be costly for routine use. The near-infrared spectroscopy (NIR) technique is a high throughput technique that involves shining a beam of infrared light on a biological sample, collecting a reflectance spectrum, and using machine learning algorithms to develop predictive algorithms. Here, we used NIR to detect DENV1 artificially introduced into whole blood, plasma, and serum collected from human donors. Machine learning algorithms were developed using artificial neural networks (ANN) and the resultant models were used to predict independent samples. DENV in plasma samples was detected with an overall accuracy, sensitivity, and specificity of 90% (N = 56), 88.5% (N = 28) and 92.3% (N = 28), respectively. However, a predictive sensitivity of 33.3% (N = 16) and 80% (N = 10) and specificity of 46.7% (N = 16) and 32% (N = 10) was achieved for detecting DENV1 in whole blood and serum samples, respectively. DENV1 peaks observed at 812 nm and 819 nm represent C-H stretch, peaks at 1130–1142 nm are related to methyl group and peaks at 2127 nm are related to saturated fatty groups. Our findings indicate the potential of NIR as a diagnostic tool for DENV, however, further work is recommended to assess its sensitivity for detecting DENV in people naturally infected with the virus and to determine its capacity to differentiate DENV serotypes and other arboviruses.

## 1. Introduction

DENV is a mosquito-borne single positive stranded RNA virus that belongs to the family Flaviviridae and genus Flavivirus [1]. It is transmitted to humans primarily by the bite of Aedes mosquitoes. *Aedes aegypti* is the primary vector, whereas *Aedes albopictus* is considered the secondary vector [2]. DENV is the world’s most common arboviral infection, with an estimated 4 billion people living in 128 countries at risk [3]. DENV infections have increased dramatically over the last 20 years, particularly in tropical countries. It is estimated that at least 390 million infections occur each year, with an estimated 300 million asymptomatic or subclinical cases [4].

There are five antigenically distinct DENV serotypes (DENV-1-5) that manifest with similar symptoms [5,6,7,8,9]. DENV is the cause of DENV fever, DENV shock syndrome, and the most severe and fatal form of the disease known as DENV haemorrhagic fever [10]. The incubation period ranges from 3 to 14 days and symptoms including joint pain, severe headache, macular rash, retro-orbital pain, and haemorrhagic manifestations can vary from 3 to 7 days [11].

Diagnosis of DENV-infected patients, which is traditionally based on their signs and symptoms, follows the WHO DENV guidelines that classifies patients into the following two categories: DENV and severe DENV. For example, a patient with DENV is defined as a person experiencing fever and two or more other symptoms, as highlighted in the WHO guidelines, i.e., pain behind the eyes and severe headache [12]. A severe DENV diagnosis consists of evidence of organ involvement and/or severe plasma leakage [12]. Diagnosis based on signs and symptoms is not always reliable, as cases between human infections differ. A study that compared clinicians’ diagnosis and WHO guidelines for diagnosis in Sri Lanka during a DENV1 epidemic found diagnosis performed by clinicians to be more specific, but less sensitive [13].

The second DENV diagnostic technique, which involves detection of anti-DENV immunoglobulin antibodies (IgM and/or IgG) using enzyme-linked immunosorbent assay (ELISA), is the most widely used technique for confirmation of DENV infection, due to its relative ease of use compared to molecular techniques [14]. However, because antibody titres can be low during initial infection, this technique has been shown to be less than 50% sensitive up to 4 days after onset of symptoms [15]. Furthermore, it has been reported in >20% patients that a second infection significantly reduces IgM titres to undetectable levels [16]. IgM can also circulate for roughly 60 days, whereas IgG circulation is lifelong, making diagnosis of any recent infections difficult [12,17]. A recent study using ELISA to detect DENV NS1 monoclonal antibodies in suspected acute phase DENV patients showed an overall sensitivity of 84.85% for DENV serotypes 1, 2, 3 and 4. However, the sensitivity for DENV3 was 75%, and that of DENV4 was 66.6% [18].

The third diagnostic technique involves the use of rapid diagnostic tests (RDTs) to detect conserved glycoprotein NS1, secreted by DENV infected cells during the acute viremic phase of infection [19]; this protein is assumed to be involved in viral replication [20]. The SD BIOLINE DENV Duo RDT Kit based on the NS1 protein was reported to be 46.8% sensitive and 87.7–95.9% specific with a sample group of 246 mixed serotype DENV patients [21]. Despite these promising findings of RDTs, results from these studies have shown that in secondary DENV infections, the NS1 window of detection is shortened due to the formation of antigen–antibody complexes with pre-existing IgG [17,22]. Secondary DENV infections are usually more severe, making this a concerning drawback. Moreover, RDTs have been reported to have low sensitivities in some areas. For example, a study in Brazil found 49% of NS1 negative samples to be positive for DENV4 [23].

Molecular techniques, such as the use of reverse transcription PCR (RT-PCR) to detect DENV viral RNA, are the most accurate and sensitive diagnostic tools for all DENV serotypes. RT-PCR has been developed and applied extensively with the implementation of various PCR primers for DENV serotypes [24]. The downside to this method is the requirement for expensive laboratory equipment and trained personnel, and the short window of opportunity for sampling, which must coincide with the viremic window of 3–5 days post infection [25,26]. Moreover, despite various PCR DENV detection protocols being developed, not all of them have reliable accuracies. An external quality assessment of the RT-PCR method for DENV diagnosis from 46 datasets taken from 37 laboratories and 27 countries was compared and only 9 laboratories were considered acceptable for diagnostics purposes (detection of >10^3^ genome equivalents/mL) and 5 for surveillance purposes (capable of serotyping DENV) [27]. More recently, Gao and colleagues used a tandem toehold-mediated displacement to detect up to six copies of dengue RNA in four serotypes [28]. However, the technique still requires specialised equipment and trained personnel to operate.

The near-infrared (NIR) technique involves the interaction of infrared radiation with biological samples to produce a diagnostic spectrum. Molecules absorb light at specific frequencies characteristic of their chemical structure [29] which implies that different biological samples with unique chemical profiles have unique absorption and reflectance properties, which can be quantified as peaks on an infrared spectrum. Moreover, NIR is non-invasive, a spectrum can be acquired in seconds, and it does not require reagents to operate.

To date, NIR has been applied to non-invasively predict the age of arbovirus and malaria vectors [30,31,32,33,34,35], differentiate mosquito species [36,37] and non-invasively detect infection status within disease vectors, including *Wolbachia* [38,39], Zika and Chikungunya viruses [40,41], *Plasmodium* parasites [42] and *Trypanosoma cruzi* parasite within Triatomine species [43]. In a more recent study, NIR has been used to estimate the time post death of *Aedes* mosquitoes that were left unpreserved in traps for 7 days [41]. Overall accuracies of NIR for all studies conducted range from 80 to 99%. However, to date, the use of NIR as a potential diagnostic tool for arboviruses has not been assessed. The main objective of this study was to determine the capability of the tool to detect DENV1 in whole blood, plasma, and serum under laboratory conditions.

## 2. Materials and Methods

### 2.1. DENV1 and Vero Cell Culture

DENV1 virus prototype strain Hawaii (KM204119) [44] was used in this study. The virus was propagated in C6/36 *Ae. albopictus* cells, maintained at 28 °C in RPMI and supplemented with 10% FBS and 1% PSG. Following three passages in C6/36 cells, virus stocks were concentrated using Ultracel-100k filters (Amicon, Tullagreen, Cork Ireland) [45] and frozen at −80 °C until further use.

Virus stocks were titrated using a modification of the CCELISA procedure of Broom et al. [46]. Briefly, virus stocks and samples were 10-fold serially diluted and inoculated onto monolayers of C6/36 cells grown in Vero cell culture media, which consisted of Roswell Park Memorial Institute Medium (RPMI), 1640 Medium (Sigma Life Sciences, Massachusetts, USA) with 5% heat-inactivated fetal bovine serum (FBS) (Thermos Fisher Scientific, Massachusetts, USA) and 1% penicillin-streptomycin flutamine solution (PSG) (Thermos Fisher Scientific, USA) and maintained at 30 °C, 5% CO_2_. After 7 days of incubation, cells were fixed in acetone: methanol (1:1) for 1 hr at 4 °C. Plates were air-dried and the antigen was detected using the following cocktail of anti-flavivirus monoclonal antibody hybridoma supernatants: 4G2 [47] and 6B-6C1:3H5 [48], at a ratio of 1:1:1, followed by horseradish peroxidase (HRP-) conjugated goat anti-mouse polyclonal antibody (DAKO, Carpinteria, CA, USA) (1:2000 in PBS-Tween 20). Antibodies bound to the cell monolayers were detected by the addition of the 3,3′,5,5′-tetramethylbenzidine (TMB) liquid substrate system for membranes (Sigma-Aldrich, Missouri, USA). The cell culture infectious dose of 50% (CCID50) was determined from the titration endpoints, as described elsewhere [49], and expressed as C6/36 CCID50/mL. The DENV1 titre used was 3.16 × 10^4^ infectious units/µL.

### 2.2. Human Blood and Serum

In total, 46 biological replicates of whole blood and 15 biological replicates of serum samples (each consisting of 150 mL of pooled human serum samples) were obtained from Australian Red Cross Lifeblood using the human ethics protocol approved by the University of Queensland (ethics approval number 2020001077). Following collection from donors, all samples were routinely tested for hepatitis B and C, HTLV I/II, syphilis HIV 1/2, and ABO/Rh antigens. Whole blood was stored at 4 °C while serum was stored at −25 °C 3–4 days prior to the experiment. To obtain plasma, whole blood from each donor was separately centrifuged at 2400 RPM for 15 min. The supernatant (plasma) was carefully titrated out into a new tube and used for the experiment. The second derivative NIR spectra of plasma can be seen in Supplementary Figure S2.

### 2.3. Spiking DENV1 in Whole Blood, Plasma, and Serum Experiment

A serial dilution of DENV1 EM-093 in Vero cell culture media at 5 dilutions (1:2, 1:10, 1:100, 1:1000 and 1:10000) was prepared by artificially adding the virus into whole blood, serum, and plasma as dilutants and these dilutions resulted in DENV1 concentrations of 1.58 × 10^4^, 3.16 × 10^3^, 3.16 × 10^2^, 3.16 × 10 and 3.16 infectious units/µL, respectively. These samples were treated as infected. Similarly, a serial dilution using Vero cell culture media at the same concentration as the virus was prepared for whole blood, plasma and serum and these samples were used as the negative control. Two µL of the mixture was aliquoted onto a microscope glass and allowed to dry for 1 hr. A total of 5 technical replicates were prepared for each biological replicate per dilution point. A total of 10, 26 and 15 biological samples were analysed for whole blood, plasma, and serum, respectively, with 8–10 biological samples being processed per day. Additional experiments to demonstrate the capacity of NIR to differentiate Vero media from Barmah Forest Virus and Ross River Virus that were conducted using blood from different donors is provided as Supplementary Materials.

### 2.4. Spectra Collection

**Figure 1:**
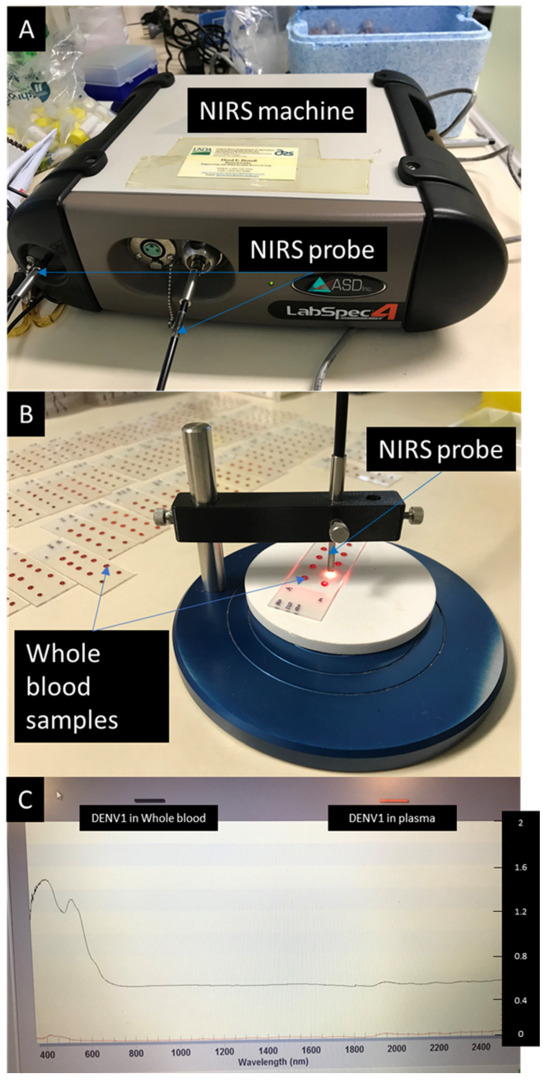
A set up of the NIR spectrometer with an external probe (**A**), the setup of samples during spectra collection (**B**) and an example of raw spectra collected from whole blood and plasma infected with DENV1 (**C**).

All samples were scanned with a LabSpec 4i near-infrared spectrometer (ASD, Malvern Panalytical, Malvern, United Kingdom). The spectrometer includes a HL-2000 halogen light source and 3 mm diameter bifurcated external fiber-optic probe, containing 6 illumination fibres with 600 microns that surround a single 600-micron collection fibre (ASD Malvern Panalytical). It has an operating wavelength of 350–2500 nm in 1 nm increments [50]. Spectra collection was carried out using RS3 software (Malvern ASD Panalytical). Optimization and baseline calibration were performed at the start of each experiment and again after every 30 min by scanning an empty space on the glass slide placed on a white reference panel. Spectra from dried spots were scanned at approximately 3 mm distance from the light source by pointing the probe down to the centre of the samples for approximately 3–5 s (Figure 1).

### 2.5. Data Analysis

Reflectance spectra were converted to absorbance using the formular Log $\frac{1}{R}$. All spectral signatures were converted from txt to csv in ViewSpecPro software (Analytical Spectral Devices Inc, Boulder, CO, USA, 1990–2017). Model screening and data analysis were conducted in JMP Pro 16 software (SAS Institute Inc., Cary, NC, USA, 1989–2021). Data were first split into plasma, serum, and whole blood, which were subsequently analysed separately. Data for each individual dilutant set were further split into training (60%), validation (20%) and test (20%) sets. To determine the appropriate machine learning algorithm for each of the data, all data underwent model screening where the following model types were screened for preliminary accuracy: decision tree [51], bootstrap forest [52], boosted tree [53], naïve Bayes [54], neural boosted [55] and support vector machines [56]. Spectral signatures from 350 to 2500 nm were used as model predictors, whereas infection status, i.e., DENV-infected samples and negative control samples, were used as the response factor. Infected samples were assigned a value of “1” and uninfected samples were assigned a value of “2”. The model with the highest predictive accuracy was chosen for developing the final prediction model. In this case, the boosted artificial neural network (ANN) model was selected for all data sets.

### 2.6. Analysis with Artificial Neural Network

Boosted ANN models were created to analyse plasma, serum, and whole blood. We used 600 spectra of whole blood, 1100 spectra of plasma and 760 spectra of serum samples. For each model developed, 60% of the spectra were used to train the model, 20% were used as a validation set and 20% were used as a test set (Table 1). To prevent self-prediction of samples (data leakage), i.e., same biological samples being used in training, validation, or tests sets, the spectra were first grouped into biological replicates. All technical replicates that arose from the same biological replicate were all used in either training, validation, or test sets. Factor reduction was utilised to identify the best wave regions of the spectra for highest predictive accuracy. This was achieved by narrowing down the entire wavelength, until a better accuracy was obtained. The final model included regions from 350 to 1099 nm for plasma samples and the entire wavelength (350–2500 nm) for whole blood and serum samples. The ANN model consisted of one layer with three TanH activation nodes iteratively boosted at a learning rate of 0.1. The number of tours for model refinement was set to five. Models were then built to predict DENV1 infection status, and the limit of detection based on the NIR spectra of whole blood, plasma and serum spiked with DENV1 or Vero media. A second order Savitzky–Golay derivative with 10 smoothing points was applied to all raw spectra to further identify peaks associated with DENV1 infection. Additional analysis to demonstrate the ability of NIR to differentiate Vero media, BFV and RRV is provided in Supplementary Table S1.

### 2.7. Test of Proportions

To determine the accuracy, sensitivity and specificity thresholds for biological replicates based on predictions of technical replicates, we used a three-sample test of proportions. A significant difference was observed between the biological samples where the predictions were in complete agreement (N = 35) (G1), biological samples where 4 of 5 technical replicates (N = 12) were in agreement (G2) and biological samples where 3 of 5 samples (N = 5) were in agreement (G3) (chi-squared = 19.55, degrees of freedom = 2, *p*-value = 5.68 × 10^−^^5^). Post hoc two sample tests of proportions were then undertaken to identify the source of the difference. When comparing those in complete agreement against those where 4 of 5 replicates agreed using a 2 populations proportions test, the value of z was -0.811 and the value of *p* was 0.41794. This result was not significant at *p* < 0.05. A similar difference was observed between samples where 4 of 5 technical replicates agreed, where the statistics for this were as follows: z = 2.3324 and *p* = 0.0198, which were also not significant at *p* < 0.05. However, when considering those in complete agreement against those where 3 of 5 replicates agreed, the following statistics were reported: z = 3.8389 and *p* = 0.00012. The result is significant at *p* < 0.05. This indicates a significant difference in accuracy between those two groups; however, one should treat the result cautiously as there was only 5 samples in G3. Based on these statistics, a biological sample was considered accurately predicted if at least 4 out of 5 of the technical replicates were predicted as the actual infection status of that sample.

## 3. Results

### 3.1. Raw Spectra of DENV1 and Vero Media in Serum, Whole Blood, and Plasma

**Figure 2:**
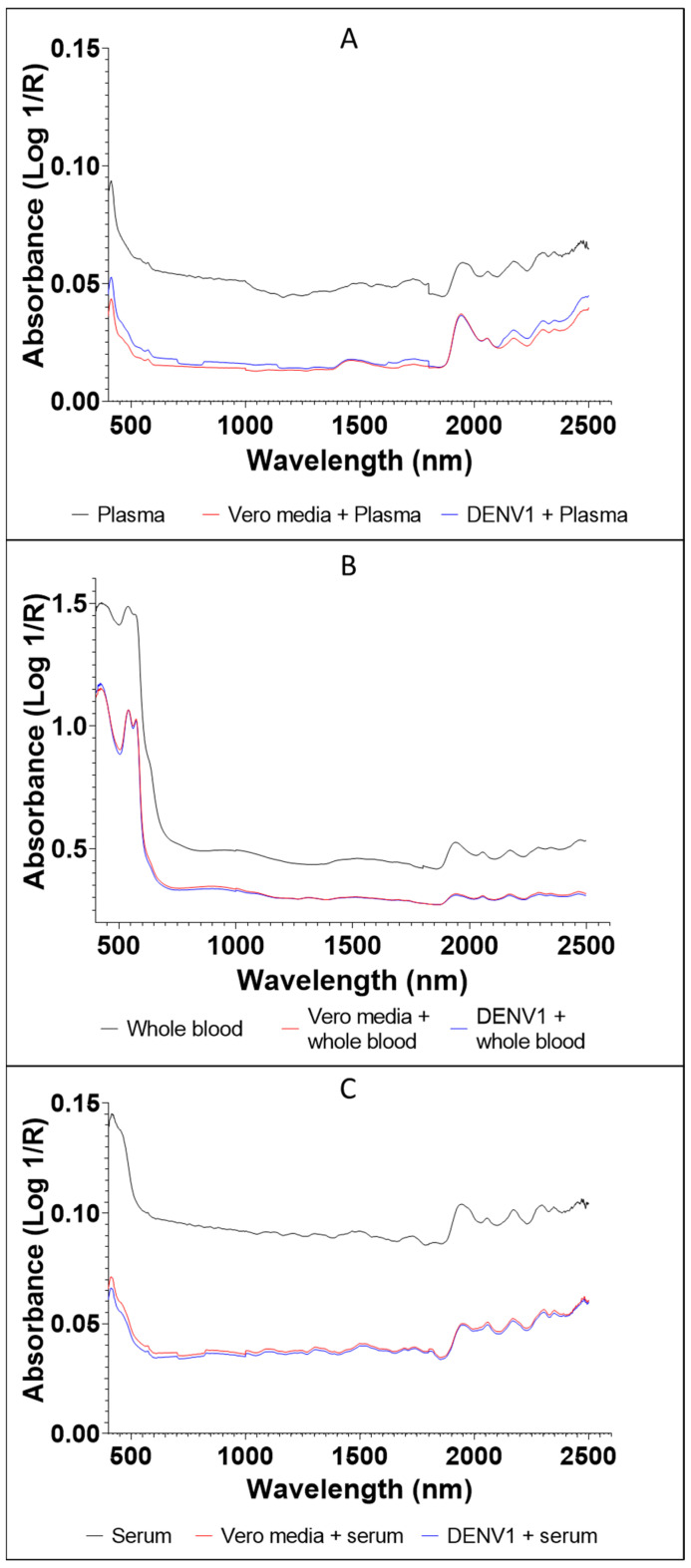
Average NIR unprocessed spectra of plasma (**A**), whole blood (**B**), and serum (**C**) alone, combined with Vero media or DENV1. Spectra of all concentrations/donor tested were averaged into a single spectrum for all samples. In total, 1100, 600 and 760 NIR spectra for plasma, whole blood, and serum samples, respectively, were collected.

**Figure 3:**
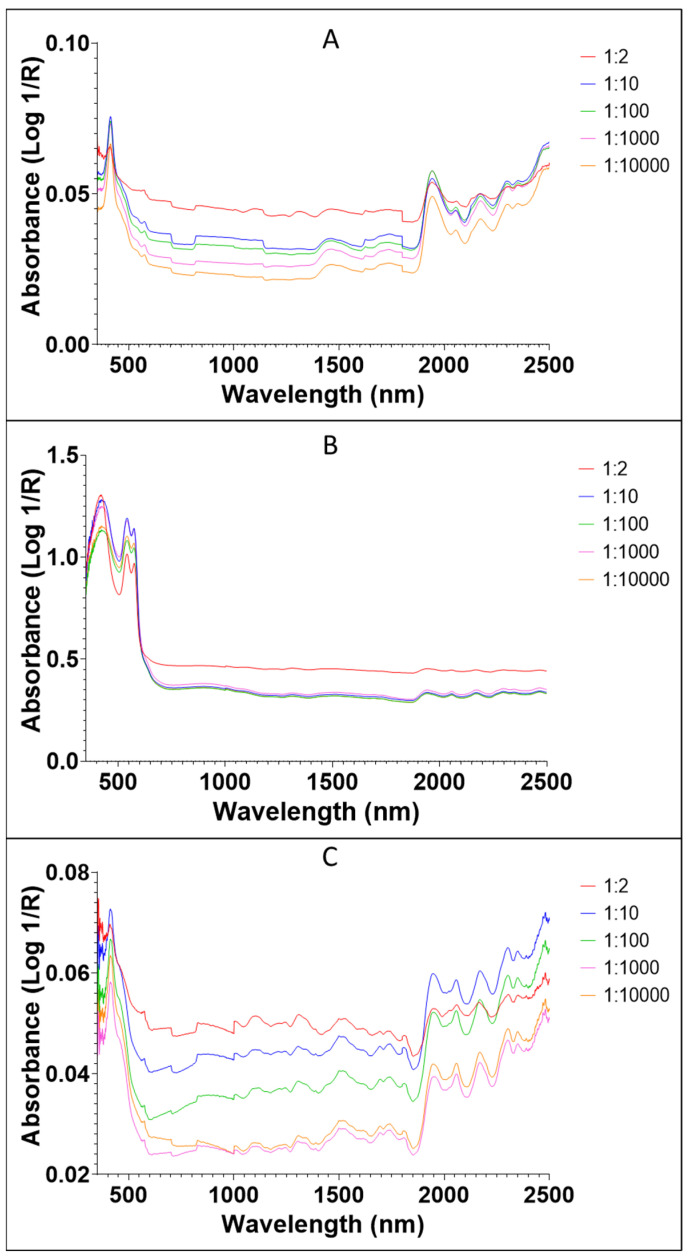
NIR spectra of all DENV1-infected samples split by concentration for plasma (**A**), whole blood (**B**) and serum (**C**). Each spectrum is an average of all spectra collected from multiple donors for the dilution shown. Initial concentration of DENV1 was 3.16 × 104 infectious unit/µL.

The average raw spectra of DENV1 and Vero media spiked into whole blood, plasma and serum are shown in Figure 2. Absorption bands were higher for the whole blood samples compared to the plasma and serum samples. This is because whole blood naturally has more water molecules and other elements compared to plasma and serum. For example, the absorption values of DENV1 in whole blood ranged from 0.35 to 1.5 (Figure 2B) compared to the absorption values of DENV1 in plasma, which ranged from 0.015 to 0.1 (Figure 2A) and the absorption values of DENV1 in serum, which ranged from 0.035 to 0.15 (Figure 2C). The average absorption values of DENV1 in serum and whole blood were generally lower than the absorption values of Vero media in serum and whole blood, whereas DENV1 in plasma, on average, absorbed more light compared to Vero media in plasma (Figure 2). When the spectra were split based on concentration, absorbance generally decreased as the virus concentration decreased for the plasma and serum samples, regardless of the donor (Figure 3). For whole blood samples, absorbance for the most concentrated sample (1:2) was higher than all other concentrations. NIR raw spectra of all donors analysed are provided in Supplementary Figures S3–S5.

### 3.2. Sensitivity, Specificity, and Accuracy of NIR for Detecting DENV1 in Plasma, Serum, and Whole Blood

Based on the test of proportion statistics obtained, to calculate sensitivity, specificity, and accuracy, the biological samples were considered to be correctly predicted if at least 4/5 of the technical replicates were correctly predicted. The results are shown for samples that were used to test the accuracy of the training set only. Table 1 shows how samples were divided between training, validation and tests sets for plasma, whole blood, and serum.

Regardless of the concentration of the virus, plasma samples were predicted with a sensitivity of 88.5% (n = 135) and specificity of 92.3% (n = 135) for those samples that were used to test the model (Table 2). However, the sensitivity and specificity for detecting DENV1 dropped to 80% (n = 50) and 32% (n = 50) for whole blood, respectively, and 33.3% (n = 75) and 46.7% (N = 75) for serum, respectively (Table 2).

### 3.3. Effect of DENV1 Concentration on Predicted Accuracy, Sensitivity and Specificity

The effect of virus concentration on the prediction accuracy, sensitivity, and specificity of NIR is shown in Table 3. The sensitivity of predicting DENV1 in plasma samples ranged from 80 to 100%. Higher concentrations, such as 1:2, 1:10 and 1:100, were predicted with a slightly lower sensitivity compared to lower concentrations. We, however, do not think that virus concentration had any effect on the sensitivity of NIR for DENV1 detection in plasma samples because sensitivity for each concentration point shown was based on 5–6 donors. For example, the sensitivity of 80% (n = 6) at 1:2 dilution was based on a correct prediction of 5/6 biological replicates. The only biological replicate that was incorrectly predicted was due to incorrect prediction of <3 technical replicates, which could be attributed to poor spectral signature collected, as opposed to the sensitivity of the technique. Based on this result, we hypothesize that NIR detected the presence of viral particles/compounds present in cell lines/ blood samples, as opposed to the virus itself, hence the observed 100% sensitivity at 1:10,000 dilution. The predictive specificity, which includes all samples scanned at various dilution points that were negative for DENV1, was 92.3% (Table 3). The model developed to predict DENV1 in whole blood was more sensitive than it was specific, and the sensitivity was 100% at 1:2 dilution, which dropped to 60–70% at 1:10 to 1:1000 and rose to 100% at 1:10000 dilution (Table 3). For serum samples, specificity across the five dilution points was 46.7%, while sensitivity dropped from 66.7% at 1:2 dilution to 33.3% at 1:10000 (Table 3).

### 3.4. Second Derivative Plots

**Figure 4:**
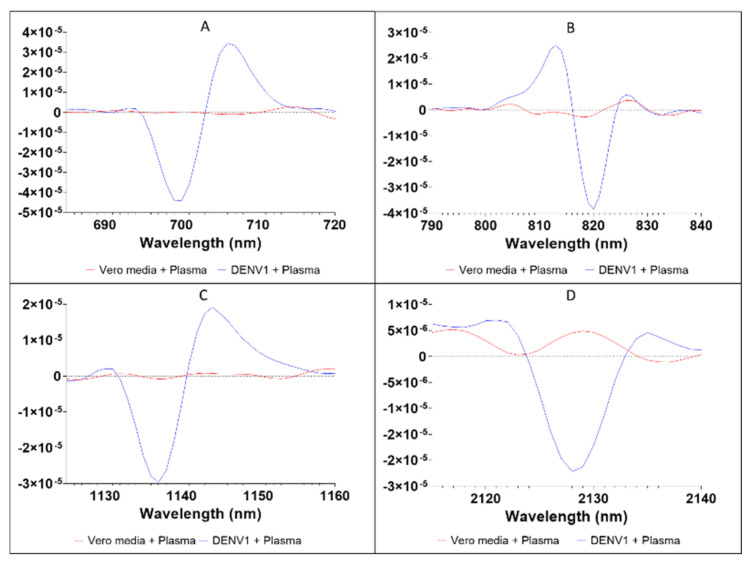
Second derivative NIR spectra of DENV1 in plasma samples. The red line indicates Vero media in plasma and the blue line indicates DENV1 in plasma. Panel (**A**) displays the wavelength range from 685 to 720 nm with DENV1 prominent peaks at 700 and 706 nm. Panel (**B**) displays the wavelength range from 790 to 840 nm with DENV1 prominent peaks at 812 and 819 nm. Panel (**C**) displays the wavelength range from 1125 to 1160 nm with DENV1 prominent peaks at 1130, 1137 and 1142 nm. Panel (**D**) displays the wavelength range from 2115 to 2140 nm with a DENV1 prominent peak at 2127 nm.

To investigate prominent peaks of DENV1 in plasma, we plotted the 2nd derivative spectra (Figure 4). We have presented results for a wider wavelength than what was analysed to determine repeated overtones. The second derivative graph shows distinct peaks for DENV1 in plasma relative to Vero media in plasma at 700, 706, 812, 819, 1130, 1137, 1142 and 2127 nm (Figure 3A–D). For whole blood and serum samples, no significant peaks could be found that were attributed to DENV1 infection. Table 4 shows the prominent absorption peaks identified from DENV1-infected samples. Peaks at 812 and 819 represent the 3rd overtone of C-H [57]. Peaks at 1130, 1137 and 1142 nm represent the 2nd overtone of C-H vibration and are indicative of the presence of methyl/methylene/oil [58]. A peak at 2127 nm represents C-H vibration and is indicative of the presence of saturated fatty acids [59].

## 4. Discussion

The objective of this study was to determine the capability of NIR coupled with machine learning algorithms to detect DENV1 in human blood samples. To achieve this, we spiked DENV1 into plasma, whole blood, and serum at different concentrations, collected NIR spectra and developed independent models to predict infection in plasma, serum, and whole blood. Our overall results show that spectral signatures collected from samples infected with DENV1 could be differentiated from samples that were used as negative control, i.e., Vero media in plasma, whole blood, or serum, albeit at varying sensitivities for each of the blood samples used. DENV1 could be differentiated from Vero media in human plasma with a sensitivity and specificity of 89% and 92%, respectively, while predictive models using whole blood and serum were 60% and 33% sensitive and 20% and 46% specific, respectively.

Model screening showed ANN boosted models were the most accurate for predicting DENV1 infection in all three blood samples used. This may indicate that the spectra of DENV1-infected and uninfected samples are not linearly distributed, but may have a pattern involved in their distribution and deep learning prediction models, such as ANN, are best suited for analysing such data [61].

The ANN models developed to differentiate DENV1-infected samples in the 350–1099 region indicate that sensitivity of NIR was highest when plasma was used, compared to whole blood and serum samples. When DENV1 was artificially introduced into plasma, regardless of the concentration of the sample, NIR detected DENV1 in plasma with 90% accuracy. NIR’s sensitivity was not affected by the concentration of the virus, with sensitivity and specificity remaining quite stable across various concentrations. It has been previously reported that people in different age groups, DENV viraemia peaks during the febrile period. During this period, viraemia has been observed to reach 10^2^–10^3^ infectious units/µL [62]. In this study, the limit of detection was 312.5 folds below the peak of DENV viraemia reported in patients, which is a significantly lower concentration. This is promising as it means that NIR could potentially detect the presence of the virus before the onset of the symptoms, prompting an early intervention. A high predictive accuracy was also reported when Raman spectroscopy was used for the detection of DENV infection in plasma, where a predictive sensitivity of 97.95% and specificity of 95.40% were observed in a pool of 17 healthy and 17 DENV-infected patients in comparison to non-structural protein 1, IgM and IgG ELISA [63].

When whole blood was used, regardless of the viral load, NIR was 80% sensitive and 32% specific. This low sensitivity achieved relative to what was observed for plasma is consistent with previous studies that used NIR to determine cholesterol levels in whole blood and blood plasma from cow’s blood. The NIR prediction accuracy was lower when whole blood was used compared to blood plasma for predicting total cholesterol, with a correlation coefficient of 0.92–0.99 for blood plasma and only 0.68 for whole blood [64]. The study suggests that the large particle size of red blood cells was responsible for low absorption and increased scattering of NIR radiation, possibly resulting in lower accuracies [51]. Similarly, in our study, lower accuracies were displayed for whole blood compared to blood plasma, which could be due to the same reason. Alternatively, low sensitivity could be related to the presence of various compounds in whole blood, which acted as confounding factors for accurate prediction of DENV1. Whole blood contains red blood cells, white blood cells and platelets, all of which absorb light at various frequencies [65]. Combined, these substances produce peaks that could have an overall effect on NIR absorbance signals for the artificially introduced virus.

The neural boosted models created for serum samples indicated a low sensitivity and specificity of 33.3% and 46.7%, respectively. This result was comparable to a study that applied Raman spectroscopy to detect DENV in blood serum, with predictive accuracies of 66% and 47% relative to IgG and IgM ELISA tests, respectively [66]. The serum samples used in our study were pooled from multiple donors and this probably introduced confounding factors, resulting in the much lower predictive sensitivity than plasma.

The spectral signatures collected from DENV1 in human plasma showed characteristic peaks at 812 and 819 nm within the 3rd overtone region, 1130, 1137, 1142 nm in the 2nd overtone region and 2127 nm within the 1st overtone region. In reference to the literature, the peaks around 1130, 1137 and 1142 nm indicate the presence of methyl/ methylene/ oil, and peaks around 2127 nm could be related to the presence of saturated fatty acids that arose from DENV1 viral particles. The peaks at 812 and 819 nm are related to 0-H within the third overtone region. Methyl groups may be present due to the synthesis of NS5 methyltransferase, which is encoded by one of the seven non-structural proteins of DENV [67]. NS5 methyltransferase is responsible for the type 1 cap formation of DENV, which is essential for mRNA capping that preserves genetic integrity during viral replication [67]. The saturated fatty acids could be from the viral envelope of the DENV particle, or a lipid bilayer derived from the C6/36 cell membrane during cell passaging [68]. The authors are uncertain whether these peaks are specific to DENV or if they are shared between viruses. If these peaks are non-specific to DENV, we believe NIR could still be a useful screening tool for differentiating between arbovirus-infected and uninfected patients. It could be coupled with molecular techniques to allow further screening to identify virus types, which would reduce the current costs associated with blanket testing using molecular techniques. Future experiments should assess the capacity of NIR to discriminate multiple arboviruses, DENV serotypes and other arboviruses and assess whether unique infrared peaks exist for the various arboviruses and if those peaks are diagnostic. We have presented data (Supplementary Materials) that indicate that the technique can potentially differentiate multiple arboviruses.

ELISA is the current gold standard diagnostic tool for DENV infection. However, due to cost, skills and time involved in processing samples, it is not suitable for large-scale sample analysis. For example, NS1 protein ELISA kits can take approximately 2–3 h of assay time and costs are approximately USD 2 per processed sample [69]. In comparison, NIR simply involves shining a beam of light onto a sample with an average spectrum acquisition time of 3 sec/sample. Following outlay costs for an instrument, it does not require sample processing procedures or reagents and can be used non-invasively. The recent diagnosis of DENV infection in plasma by Raman spectroscopy produced comparable accuracies to our results, but with relatively longer spectral acquisition time (30 sec per sample) [63].

## 5. Conclusions

Our findings indicate the potential of the future application of the NIR technique for DENV detection in human samples. Subsequent research should assess the capacity of the technique to detect and quantify DENV in naturally infected human subjects and assess an appropriate sample preparation protocol for field adoption. It is envisaged that once robust diagnostic models have been developed and validated in multiple epidemiological settings, NIR could be used to analyse thousands of samples in a day without any skilled expertise, reagents, or sample processing procedures and could be applied to reduce outbreaks through timely detection of infections. Existing portable spectrometers can significantly reduce the outlay costs of the spectrometer used in this study, but these portable spectrometers need to be tested in the field first.

## Figures and Tables

**Table 1:** The flow of information from data collection to analysis, including the number of samples used for training, validation, and testing models. N refers to the number of biological replicates and *n* refers to the total number of NIR spectra (technical replicates of the biological replicates) collected/analysed for each data set. The training, validation and test sets were split according to biological replicates. At the model screening step, machine learning techniques, such as decision tree, bootstrap forest, boosted tree, naïve Bayes, neural boosted, support vector machines were applied. T represents training set, V represents validation set and t represents test set.

Raw Reflectance Values Converted to Absorbance	Raw Reflectance Values Collected								
	$\mathrm{Log}\;\frac{1}{R}\;\mathrm{Formula}\;\mathrm{Applied}$								
	All NIR Spectra; N = 53, n = 2360								
Data separated by blood type	Plasma NIR spectra; N = 28, *n* = 1100			Whole blood NIR spectra; N = 10, *n* = 500			Serum NIR spectra; N = 15, *n* = 760		
Samples split into training, validation, and test set	T N = 16, *n* = 650	V N = 6, *n* = 180	t N = 6, *n* = 270	T N = 14, *n* = 300	V N = 3, *n* = 100	t N = 3, *n* = 100	T N = 9, *n* = 460	V N = 3, *n* = 150	t N = 3, *n* = 150
Data processing	Prediction model screening: decision tree, bootstrap forest, boosted tree, naïve Bayes, neural boosted, support vector machines								
	Wavelength screening with ANN (350–2500 nm)								
	Neural boosted model optimisation								
Combined technical replicates into single predictive outcome	Plasma t N = 6, *n* = 270			Whole blood t N = 2, *n* = 100			Serum t N = 3, *n* = 150		

**Table 2:** A summary of number of donors, NIR spectra collected, distribution of samples in the training, validation, and test sets of neural models, and the sensitivity, specificity, and accuracy of the test set.

Blood	No. of Donors	No. Conc. Tested Per Donor	No. Spectra Collected Per Conc	No. Spectra Collected	No. Spectra in Training Set	No. Spectra in Validation Set	No. Spectra in Test Set	%Sen (n)	%Spec (n)	%Acc (n)
Plasma	26	5	5	1100	650	180	270	88.5 (135)	92.3 (135)	90.4 (270)
Whole blood	10	5	5	500	300	100	100	80 (50)	32 (50)	56 (100)
Serum	15	5	5	760	460	150	150	33.3 (75)	46.7 (75)	40 (75)

No.—number. %Sen—% sensitivity. %Spec—% specificity. %Acc—% accuracy.

**Table 3:** A table summary of the sensitivity and specificity of ANN models developed to predict DENV1 in plasma, whole blood and serum and applied on the test set.

Blood Type		Dilution Factor				
		1:2	1:10	1:100	1:1000	1:10000
Plasma	%Sensitivity (n)	83.3 (35)	80 (25)	80 (25)	100 (25)	100 (25)
	%Specificity (n)	92.3 (135)				
Whole blood	%Sensitivity (n)	100 (10)	60 (10)	70 (10)	70 (10)	100 (10)
	%Specificity (n)	32 (50)				
Serum	%Sensitivity (n)	66.7 (15)	33.3 (15)	0 (15)	33.3 (15)	33.3 (15)
	%Specificity (n)	46.7 (75)				

**Table 4:** A summary of prominent wavelengths identified for DENV1 in plasma and their indicative functional groups from the literature [57,58,59,60].

Wavelength(nm)	Functional Group/Vibration	Representation from the Literature
700	Visible light region	Red visible light
706		
812	3rd overtone C-H	Lipids
819		
1130	2nd overtone C-H	Methyl/methylene/oil
1137		
1142		
2127	C-H stretch	Saturated fatty acids

## Data Availability

All data supporting these findings are available through the corresponding author institutions.

## Supplementary Materials

The following supporting information can be downloaded at: https://www.mdpi.com/article/10.3390/v14102248/s1, Figure S1: The average NIR spectra of a BFV, RRV and Media in human plasma sample; Figure S2: The second derivative NIR spectra of a human plasma sample; Figure S3: NIR spectra of all DENV1 infected plasma samples split by donor; Figure S4: NIR spectra of all DENV1 infected serum samples split by donor; Figure S5: NIR spectra of all DENV1 infected whole blood samples split by donor; Table S1: summary of the accuracy of differentiating Vero media cells, BFV and RRV in plasma. Reference [70] is cited in the supplementary materials.
