# Supplementary material for: First Report of the Detection of DENV1 in Human Blood Plasma with Near-Infrared Spectroscopy
Source: Viruses. 2022 Oct 13;14(10):2248. doi: 10.3390/v14102248 (PMC9609583; doi:10.3390/v14102248)
Supplement: Supplementary file 1 [file viruses-14-02248-s001.zip › viruses-1941855-supplementary.pdf]

NIRS and machine learning differentiation of Ross River virus, Barmah Forest virus and Vero Media cells

To show that NIR coupled with ANN can differentiate between different arboviruses and Vero media cells, Barmah Forest virus (BFV) strain BH2193, GenBank No. NC_001786) and Ross River virus (RRV) QML1 strain (GenBank No. GQ433354) in Vero media cells were added into human blood plasma. The virus strains were passaged three times in Vero cells, maintained at 37°C in RPMI and supplemented with 10% FBS and 1% PSG. Following three passages in Vero cells, virus stocks were concentrated using Ultracel-100k filters (Amicon, Tullagreen, Cork Ireland) (Hugo, Stassen et al. 2019) and frozen at -80°C until further use. One vial of the viral stocks was thawed to determine virus titre by 50% tissue culture infectious dose (TCID50/ml) on Vero cells as described (Sudeep et al., 2019). Briefly, virus stocks were 10-fold serially diluted and 100µl of diluted virus were inoculated onto monolayers of Vero cells grown in 96 well plates in cell culture media and maintained at 37°C, 5% CO_2. Ninety-six hours later, cells were fixed with 3.7% formaldehyde, stained with 1% crystal violet for 1 hour, washed in tap water and dried. The TCID50 was determined from titration endpoints as described elsewhere (Charretier et al., 2018) and expressed as the Vero cell TCID50/mL. The RRV titer used was TCID 50/mL= $10^{7.5}$ mL and Barmah Forest virus titer used was infectious unit/mL = $10^{6.8}$ mL.

NIR spectra followed the same procedure as that of DENV1 (Refer to materials and methods for DENV1 in the main document). A total of 20 biological replicates of plasma were used. ANN model to differentiate Vero media, BFV and RRV was generated in JMP Pro 16. A total of 600 NIR spectra were used in ANN model generation. The data set was split into training and validation set at a ratio of 80% in the training set and 20% in the validation set. Wavelength

range used in the model was 350-2500 nm. The Neural Networks consisted of two layers with 3 TanH, 1 linear and 1 gaussian activation nodes for the first layer and 3 TanH, 1 linear and 1 gaussian activation nodes for the second layer. Boosting of the model was set at a learning rate of 0.1. The number of tours for model refinement was set to 500. The results are shown as a confusion matrix in supplementary table 1. In both training and validation sets, prediction rates for BFV, RRV for both BFV and RRV were above 81.3% indicating the ability of NIR coupled to distinguish between BFV, RRV and Vero media cells. A lower prediction accuracy for negative control samples could be due to its presence in all three BFV and RRV.

Table S1. A summary of the accuracy of differentiating Vero media cells, BFV and RRV in plasma. Training and validation set accuracies are shown. n represents the number of technical replicates used in the ANN model.

Training set				Validation set			
Actual	Predicted rate (n)			Actual	Predicted rate (n)		
	**Vero media**	**BFV**	**RRV**		**Vero media**	**BFV**	**RRV**
**Vero media**	0.713 (114)	0.106 (17)	0.181 (29)	**Vero media**	0.725 (29)	0.050 (2)	0.225 (9)
**BFV**	0.106 (17)	0.838 (134)	0.056 (9)	**BFV**	0.075 (3)	0.875 (35)	0.050 (2)
**RRV**	0.175 (28)	0.013 (2)	0.813 (130)	**RRV**	0.075 (3)	0.025 (1)	0.900 (36)

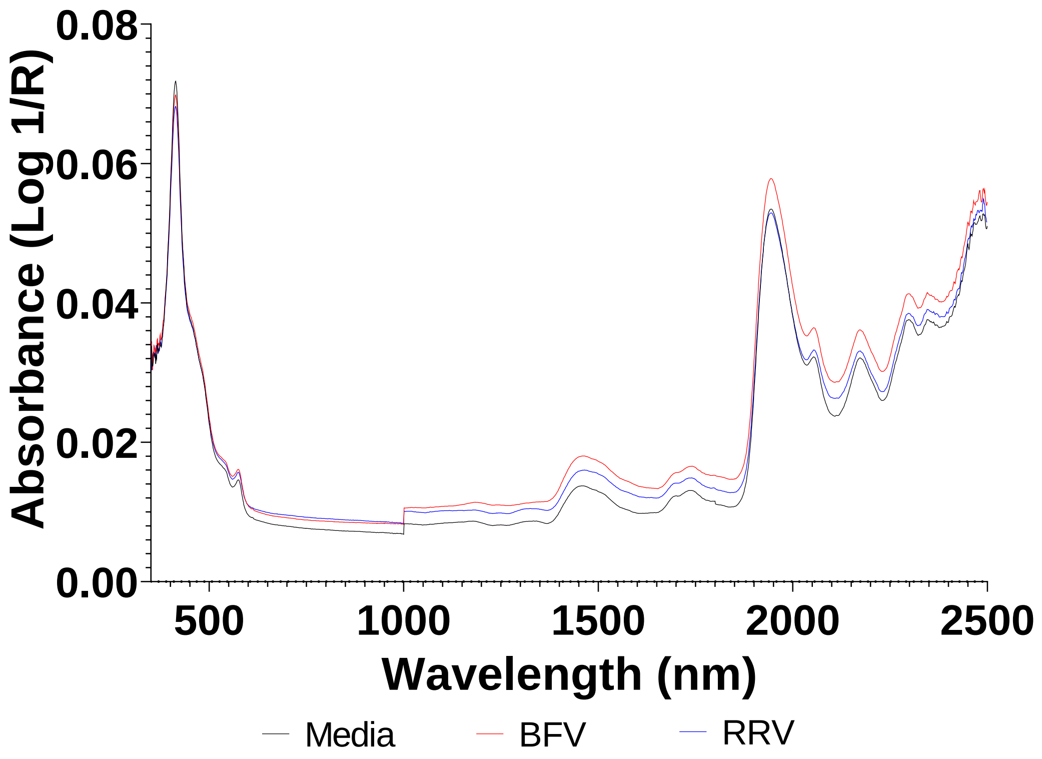

Figure S1. The average NIR spectra of a BFV, RRV and Media in human plasma sample. Each line is an average of 20 biological replicates.

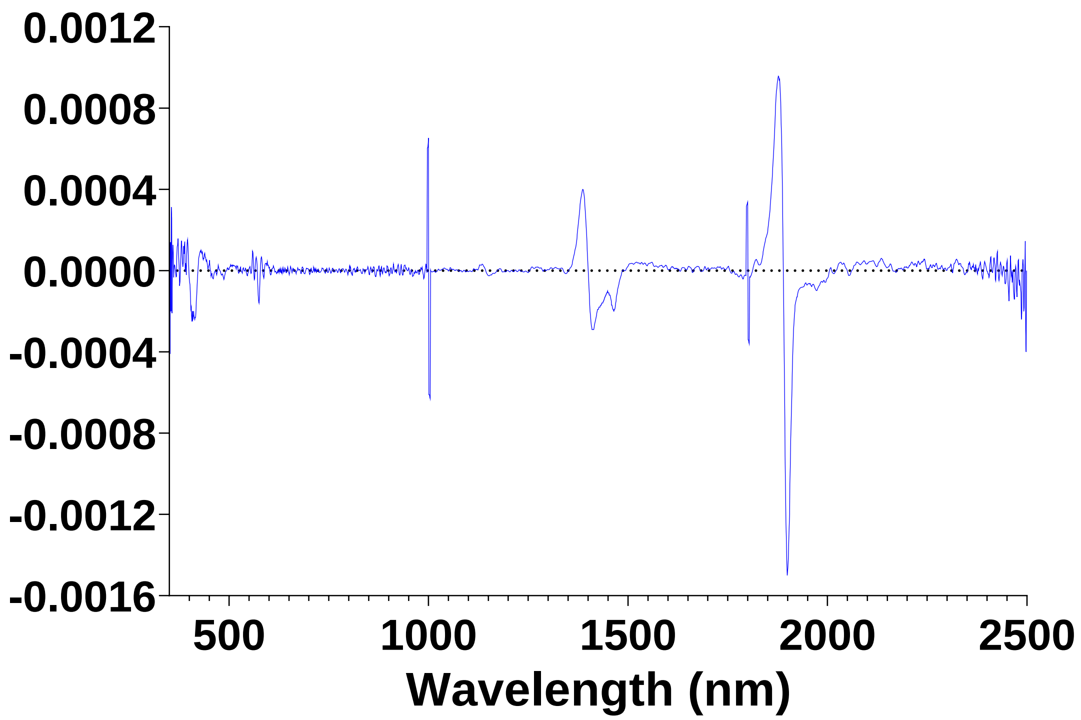

Figure S2. The second derivative NIR spectra of a human plasma sample. The spectra is an average of 10 technical replicates from a single donor.

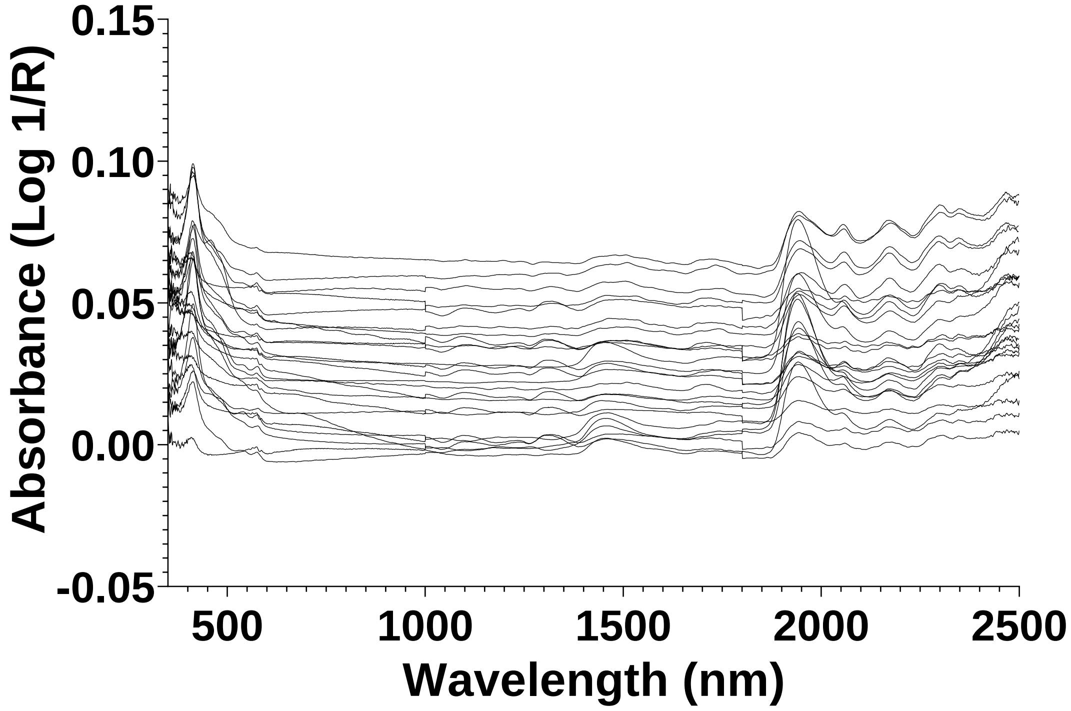

Figure S3. NIR spectra of all DENV1 infected plasma samples split by donor. Each line represents a single donor. A total of 23 donors are included in the graph.

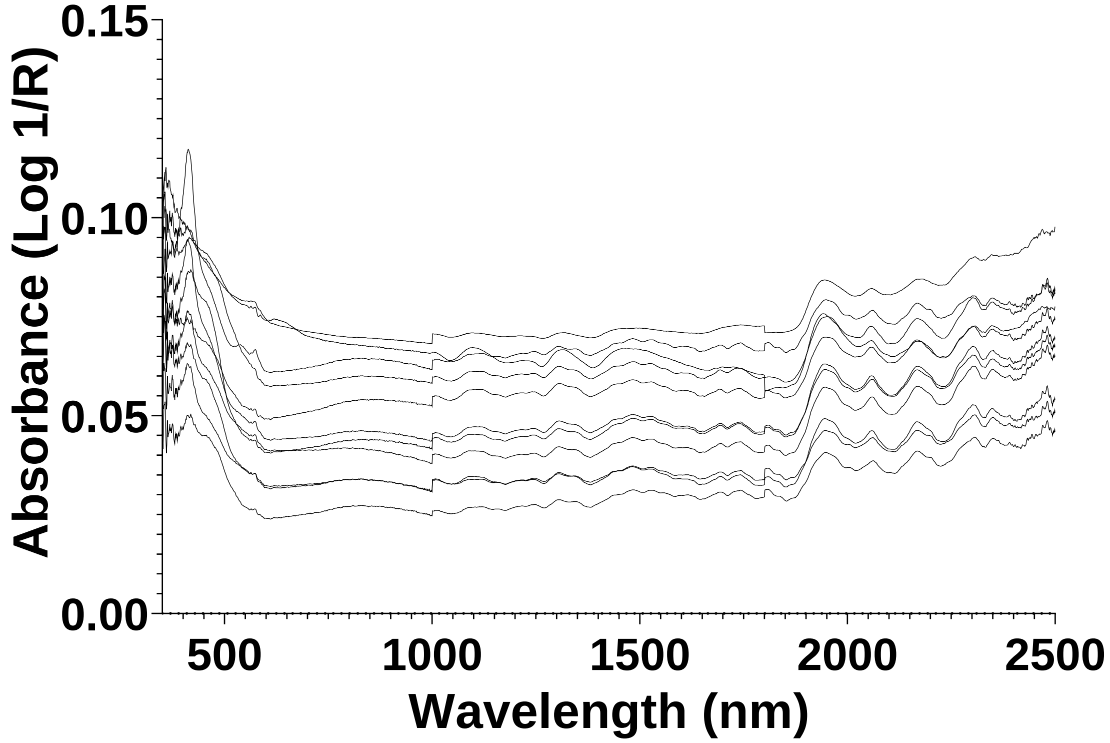

Figure S4. NIR spectra of all DENV1 infected serum samples split by donor. Each line represents a single donor. A total of 11 donors are included in the graph.

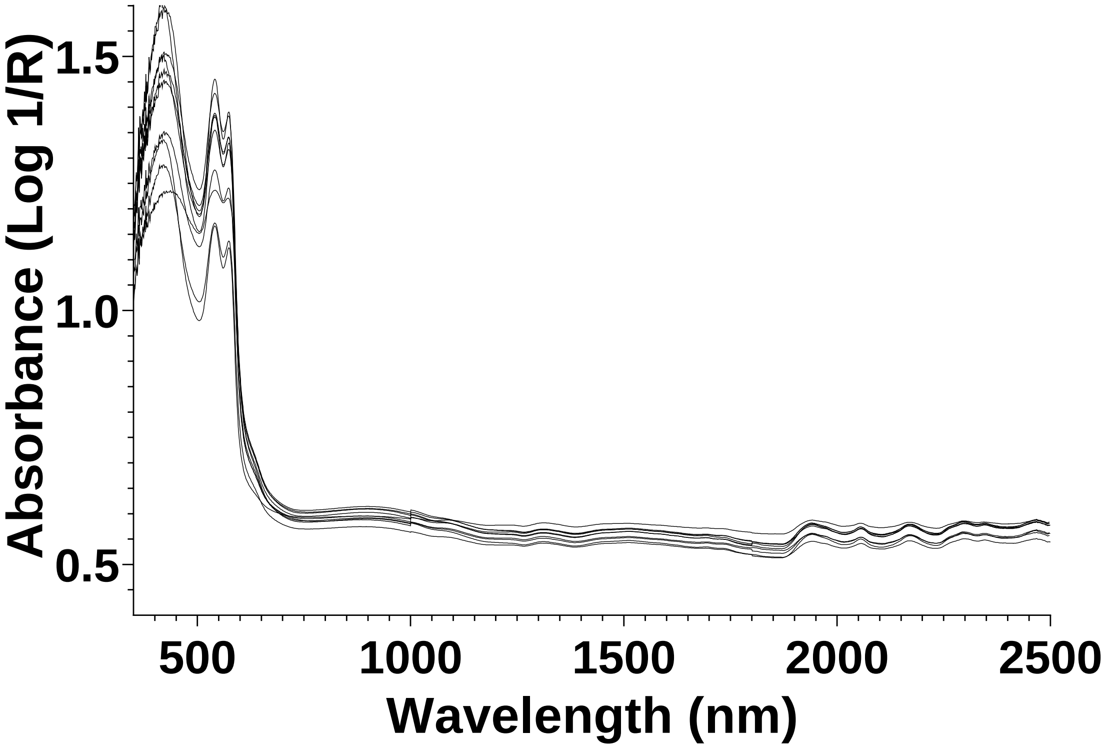

Figure S5. NIR spectra of all DENV1 infected whole blood samples split by donor. Each line represents a single donor. A total of 10 donors are included in the graph.

References

1. Charretier, C.; Saulnier, A.; Benair, L.; Armanet, C.; Bassard, I.; Daulon, S.; Bernigaud, B.; de Sousa, E.R.; Gonthier, C.; Zorn, E.; et al. Robust real-time cell analysis method for determining viral infectious titers during development of a viral vaccine production process. *J. Virol. Methods* **2018**, *252*, 57–64.

2. Sudeep, A.B.; Vyas, P.B.; Parashar, D.; Shil, P. Differential susceptibility & replication potential of Vero E6, BHK-21, RD, A-549, C6/36 cells & Aedes aegypti mosquitoes to three strains of chikungunya virus. *Indian J. Med. Res.* **2019**, *149*, 771–777.
